# Bioengineered 3D Ovarian Models as Paramount Technology for Female Health Management and Reproduction

**DOI:** 10.3390/bioengineering10070832

**Published:** 2023-07-13

**Authors:** Julieta S. Del Valle, Susana M. Chuva de Sousa Lopes

**Affiliations:** 1 Department of Anatomy and Embryology, Leiden University Medical Center, 2333 ZC Leiden, The Netherlands; 2 Department for Reproductive Medicine, Ghent University Hospital, 9000 Ghent, Belgium

**Keywords:** ovarian organoid, artificial ovary, tumoroids, reproductive biology, in vitro folliculogenesis, tissue engineering, personalized medicine, pluripotent stem cells, ovarian cancer

## Abstract

Ovarian dysfunction poses significant threats to the health of female individuals. Ovarian failure can lead to infertility due to the lack or inefficient production of fertilizable eggs. In addition, the ovary produces hormones, such as estrogen and progesterone, that play crucial roles not only during pregnancy, but also in maintaining cardiovascular, bone, and cognitive health. Decline in estrogen and progesterone production due to ovarian dysfunction can result in menopausal-associated syndromes and lead to conditions, such as osteoporosis, cardiovascular disease, and Alzheimer’s disease. Recent advances in the design of bioengineered three-dimensional (3D) ovarian models, such as ovarian organoids or artificial ovaries, have made it possible to mimic aspects of the cellular heterogeneity and functional characteristics of the ovary in vitro. These novel technologies are emerging as valuable tools for studying ovarian physiology and pathology and may provide alternatives for fertility preservation. Moreover, they may have the potential to restore aspects of ovarian function, improving the quality of life of the (aging) female population. This review focuses on the state of the art of 3D ovarian platforms, including the latest advances modeling female reproduction, female physiology, ovarian cancer, and drug screening.

## 1. Introduction

**Figure 1:**
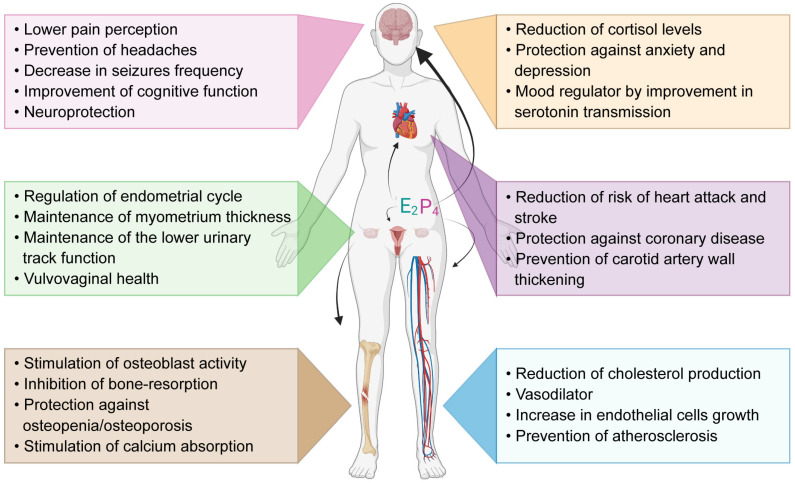
Functions of ovarian produced hormones estrogen E2 and progesterone P4 in the human body. E2 and P4 have functions on the nervous system, cardiovascular system, reproductive system and bone structure. Parts of this figure were created using https://www.biorender.com/ (accessed on 25 April 2023).

This decade, ovarian research in humans has increased in significance and scope due to an increased access to human ovarian samples (from healthy and pathology-carrier donors as well as from abortion material) [1,2,3], the growing use of innovative technologies (omics, bioengineering, computational tools) [4,5] and advances in stem cell biology [6]. The significance of ovarian research relies on the pivotal function that this organ has in the female body (Figure 1). The ovaries play vital roles in female physiology, serving as endocrine glands and reproductive organs [7], ensuring the monthly maturation of ovarian follicles and the ovulation of a fertilizable oocyte. Furthermore, the ovaries produce androgens, estrogens, and progesterone, not only necessary to allow implantation of the embryo in the receptive uterus during pregnancy, but also important to protect against osteoporosis, cardiovascular disease, and cognitive decline [8,9,10,11].

The ovarian follicle is the functional unit of the ovary, consisting of one oocyte (or egg) surrounded by one or more layers of granulosa cells and separated from the theca cell layer by an extracellular matrix (ECM)-rich basement membrane [12]. Importantly, the ovary also contains a stromal compartment, formed by various cell types and structures necessary to support the optimal dynamics of follicular maturation and remodeling during each ovarian cycle [13]. This compartment includes different types of immune cells, peripheral nerve cells, endothelial cells, perivascular cells, smooth muscle cells and ovarian surface epithelium (OSE) cells [13,14,15].

Ovarian research has several applications, such as increasing fertility preservation options and understanding ovarian cancer. To increase fertility preservation options, researchers use ovarian cortex, follicles or oocytes to improve the culture conditions necessary to induce gamete maturation in vitro, whereas research on ovarian cancer focusses on the ovarian stroma. Two components of the ovarian stroma, the OSE and ECM, are thought to play crucial roles in ovarian cancer. Evidence from multiple organ models has shown that the accumulation of ECM proteins, such as collagens, in the stromal compartment can result in tissue fibrosis, creating a more receptive environment for the growth of solid tumors [16]. Moreover, both the OSE and the fallopian tube epithelium (FTE) are considered to be the origin of high-grade serous ovarian cancer (HGSC), the most common and lethal form of ovarian cancer [17].

Over the years, both animal models and various in vitro 2D culture methods have been used to study aspects of the different ovarian functions, however these approaches lack translation to human reproductive physiology, and, more importantly, they do not recapitulate the complex dynamics of the human ovary [18]. Recent progress in 3D culture and bioengineering has enabled the development of in vitro human-based culture systems that mimic aspects of ovarian tissue organization and cell behavior more faithfully.

A groundbreaking innovation was the generation of tissue organoids, miniaturized in vitro versions of in vivo organs that are able to recapitulate (at least one) morphological or functional feature [19]. Organoids can start from single cells isolated from tissue or from stem cells, such as liver [20] or intestinal [21] organoids; or originate from multicellular aggregates [22,23]. In the latter case, the tissue sample or stem cells used to derive organoids can contain different cell types, such as stromal cells, peripheral nerve cells, vasculature and/or immune cells [24,25]. The inclusion of several cell types within the same organoid allows better self-organization and in vitro representation of the cellular interactions that occur under physiological and pathological conditions.

**Figure 2:**
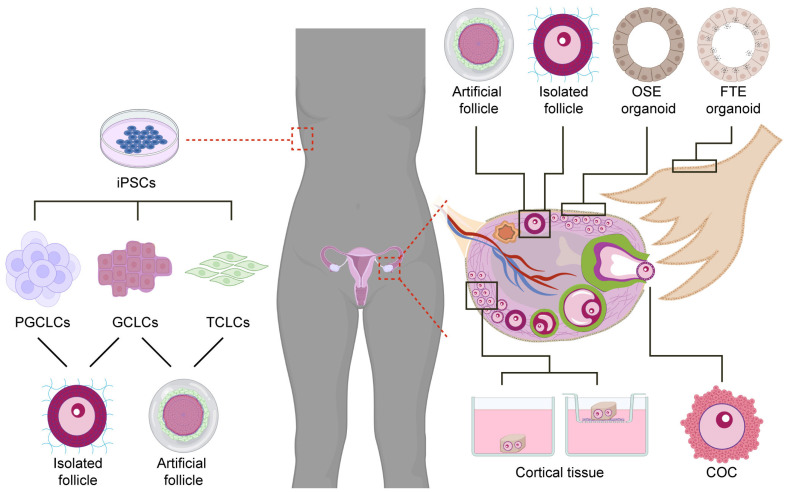
Different types of human ovarian organoids. Ovarian organoids can be isolated from different cell types and tissues of the ovary (right side) or differentiated from induced pluripotent stem cells (iPSCs) (left side). Cortical tissue containing unilaminar (primordial/primary) follicles cultured in media or air–liquid interface; complex-oocyte-cumulus (COC) cultured in medium drops; artificial follicles formed by reaggregating isolated granulosa and theca cells; isolated pre-antral follicles embedded in hydrogel and cultured further; ovarian surface epithelium (OSE) organoids generated from the OSE are often compared with fallopian tube epithelium (FTE) organoids, which are non-ovarian organoids. iPSCs cells isolated from somatic tissue can be differentiated into primordial germ cell-like cells (PGCLCs), granulosa cell-like cells (GCLCs), and theca cell-like cells (TCLCs), that can potentially be reaggregated to generate either isolated follicles or artificial follicles for further growth and maturation. Parts of this figure were created using https://www.biorender.com/ (accessed on 25 April 2023).

This review uses the term “ovarian organoids” (also known as “artificial ovaries” or “miniovaries”) to refer to any 3D structure formed by cells that display aspects of in vitro organization and functionality similar to that observed in any part of the ovary (Figure 2). The source of cells to generate ovarian organoids can either be the ovary (small cortical tissue biopsies, isolated follicles, female fetal gonads, primary cells isolated from ovarian tissue) or alternatively pluripotent stem cells (PSCs). Differentiation protocols starting from PSCs are being developed for both the germline and gonadosomatic lineages [6,26]. This review primarily focuses on advancements in ovarian research using ovarian organoids from the last five years. While studies involving animal models such as mice, rats or non-human primates have also been considered due to their significant impact, priority has been given to human-based research.

## 2. Ovarian Organoids to Study Oocyte Formation and Follicular Assembly

In humans, the first step of gametogenesis is the formation of primordial germ cells (PGCs). The PGCs migrate from the dorsal part of the yolk sac to the place where subsequently the bipotential gonads will form. Once in the gonads, some PGCs continue mitotic proliferation, while others upregulate DDX4 and DAZL to become oogonia/gonocytes. From the second half of fetal development, progressively more oogonia enter meiosis, arrest in diplotene (dictyate) of meiotic prophase I and become surrounded by a single layer of flat granulosa cells, giving rise to primordial follicles [27,28]. At birth, the pool of (resting) primordial follicles forms the finite ovarian reserve available during the entire reproductive lifespan.

In contrast to humans, in mice it is already possible to recapitulate female gametogenesis in vitro solely using mouse pluripotent stem cells (mPSCs). Yoshino and colleagues demonstrated that functional metaphase II (MII) oocytes able to be fertilized and to produce healthy offspring could be obtained in vitro using mPSCs as source for both the oocytes and the ovarian somatic cells [29]. In that work, mouse primordial germ cells like cells (mPGCLCs) and FOXL2+ ovarian gonadal somatic cells-like cells (mFOSLSs) were first differentiated in parallel and subsequently aggregated. After co-culture, the mPGCLCs upregulated oocyte-maker DDX4, entered meiosis and formed oocytes. In the mPSC-derived ovarian organoids, primordial follicles emerged and developed to the secondary stage. Interestingly, although no antral follicles formed, the oocytes retrieved were able to mature into MII oocytes. Those were then fertilized in vitro (30.2% fertilization rate) and a few gave rise to live offspring (11 pups) that developed into adult mice [29]. This suggested that the intermediate (antral) follicular stage is not necessary for oocyte maturation in mice.

In humans, it is possible to differentiate human PGCLCs (hPGCLCs) from human PSCs, but the efficiency of these protocols is highly variable depending on the PSC lines used [30,31,32]. Yamashiro and colleagues introduced a differentiation protocol whereby hPGCLCs matured further into meiosis but failed to progress through meiotic recombination [33]. In this protocol, PSCs were first differentiated into hPGCLCs and subsequently aggregated with mouse fetal ovarian somatic cells to generate ovarian organoids. After a long period of co-culture (77 days), the human cells expressed oogonia markers, such as DDX4 and DAZL. One plausible explanation for the observed meiotic arrest could be a mismatch between the hPGCLCs and the developmental stage of the mouse ovary used for the aggregation. In humans, follicular formation using PSCs to differentiate both the germ and somatic compartment in vitro remains to be demonstrated.

In addition to studies on human gametogenesis and follicular formation, stem cell-derived ovarian organoids are also being used as disease model to investigate causes of infertility. In a case report, researchers recently tested whether infertility in monoamniotic (MA) monozygotic twins, affecting only one of the twins, was due to the inability of that twin to generate PGCs during embryonic development [34]. For this, Pandolfi and colleagues differentiated induced PSCs (iPSCs) obtained from two infertile individuals diagnosed with primary ovarian insufficiency (POI) and their respective MA twins into hPGCLCs, but there were no significant differences between hPGCLCs obtained from the infertile and fertile twin in the two pairs studied [34]. They then tested whether iPSCs from each twin could generate amniotic sac-like structures, since both twins shared one single amnion during gestation and human PGCs are thought to originate from the amnion [35]. Regardless of the fertility condition of the donors, all iPSC lines formed both amniotic ectoderm-like cells and hPGCLCs. These results suggested that those MA twins may have shared the pool of PGCs, but these may have migrated disproportionately to the fertile twin. A severe reduction in the pool of specified PGCs would prevent the infertile twin from generating a sufficient number PGCs to overcome POI as a young adult.

## 3. Ovarian Organoids to Study Follicular Growth and Oocyte Maturation

### 3.1. Ovarian Cortex Tissue

In the adult ovary, the largest population of follicles consists of (resting or dormant) primordial follicles [12]. These follicles are located in the ovarian cortical region, rich in ECM proteins [36], within one millimeter from the OSE. Primordial follicles in the ovarian cortex tissue have the highest survival rate after cryopreservation-thawing procedures, making them adequate for fertility preservation [2]. However, there are certain groups of patients, such as oncological patients with blood-related cancer types and transmasculine individuals undergoing sex reassignment surgery, that cannot benefit from fertility preservation techniques that rely on the reintroduction (transplantation) of cryopreserved ovarian cortex tissue. An experimental approach using cryopreserved ovarian cortex tissue, that could give these patients the opportunity to become biological parents, would be to culture this tissue, triggering follicular maturation in vitro to ultimately obtain MII oocytes that could be fertilized.

Although primordial follicles present in the ovarian cortex survive cryopreservation-thawing procedures, several studies have reported that their isolation from the surrounding stroma compromises their viability [37,38]. Moreover, the use of cryopreserved-thawed human ovarian cortex tissue for in vitro folliculogenesis remains an immense challenge [39]. Despite the technical limitations using cryopreserved tissue, two studies starting from freshly isolated human ovarian cortex tissue reported the generation of MII oocytes grown from unilaminar (primordial/primary) follicles in vitro [40,41]. McLaughlin and colleagues designed a multistep culture system: in the first step, 0.5 mm^3^ ovarian cortex fragments from cisgender women undergoing cesarian-section were cultured for 8 days; in the second step, secondary follicles were isolated from the in vitro-grown ovarian tissue pieces and cultured in the presence of 100 ng/mL of Activin A for an additional 8 days, resulting in the formation of antral follicles; and in the final step, cumulus-oocyte complexes (COCs) were harvested from the in vitro-grown antral follicles and used for in vitro maturation to obtain MII oocytes [40]. By comparison, Xu and colleagues cultured smaller ovarian cortex fragments (0.014 mm^3^) in the first step and used 100 ng/mL of AMH, instead of Activin A, in the second step to induce follicular growth up to the pre-antral stage [42], followed by 100 ng/mL neutralizing anti-human AMH antibody to allow formation of the antral cavity [43]. Both Activin A and AMH are ligands that belong to the TGF-β superfamily, but interestingly they share neither the TGF-β signaling pathway transmembrane receptors, nor the used intracellular SMADs [44]. Regardless of the successful outcome, the maturation rate reported in these two studies was low (the total number of matured MII oocytes was 9 and 3, respectively), considering that they started from 160 [40] and 40 [41] fresh ovarian cortex fragments. Finally, it remains unclear whether the obtained MII oocytes could be fertilized and undergo preimplantation development.

Ovarian cortex tissue fragments can be cultured on top of coated inserts on air–liquid interface [45]. It has been shown that culturing freshly isolated human ovarian cortex tissue for 7 days on laminin-coated inserts, particularly LN521, resulted both in follicular growth (to secondary follicles) and steroidogenesis [46]. In addition, 3D-printed microporous hydrogel scaffolds seeded with mouse follicles and subsequently implanted into surgically sterilized mice were able to successfully restore fertility [47], showcasing the potential of 3D-printed bioprosthetic scaffolds in reproductive biology. Moreover, advanced bioengineered culture platforms can now better mimic the microenvironment of the ovary and the female reproductive tract, for instance using microfluidics technology [48,49]. For example, interconnected culture wells can be linked to pumps that allow a constant supply of fresh media carrying growth factors and nutrients while removing waste products. Microfluidics technologies have proven beneficial to create a more physiological microenvironment for the culture of several female reproductive tissues (ovary, fallopian tube, uterus, cervix and liver) with sustained circulation between them for a period of 28 days [50]. Recently, Del Valle and colleagues investigated whether culturing human cryopreserved ovarian cortex fragments (1 mm^3^) under dynamic flow conditions could improve the maturation rate of unilaminar follicles to secondary follicles after 8 days of culture [51]. This study followed the first culture step described by McLaughlin and colleagues [40], but used cryopreserved ovaries from transmasculine individuals on hormone replacement therapy for several years instead. They showed that neither a low flow rate (0.1 mL/min) nor a high flow rate (0.5 mL/min) were beneficial for the maturation to secondary follicles [51]. The secondary follicles obtained presented a disorganized layer of granulosa cells, often with a peripherally located oocyte. Furthermore, the highest number of apoptotic cells was detected within the stromal compartment of ovarian fragments cultured under dynamic conditions. These results may be explained by the fact that continuous flow may prevent the local accumulation of paracrine signals important to promote follicular activation and growth in the poorly vascularized ovarian cortex tissue [52]. In addition, a negative influence from the androgen therapy on the follicular quality cannot be excluded.

### 3.2. Isolated Antral Follicles

In contrast to unilaminar follicles, secondary/pre-antral follicles can be successfully isolated from the cortical stromal tissue and subsequently cultured using hydrogels resulting in further maturation [53,54]. Alginate has been the most commonly used hydrogel to culture pre-antral follicles due to biocompatibility, stability during culture and easy encapsulation [55]. Alginate-encapsulated secondary follicles were able to mature up to the MII oocyte stage [56], as well as to produce a hormonal profile comparable to that observed in the follicular phase [57].

The use of hydrogel scaffolds containing bioactive peptides to recapitulate the ovarian microenvironment has become more popular. For example, biomimetic polyethylene glycol-based hydrogel functionalized with ECM-binding peptides seems to improve follicle survival, development of the antral cavity and oocyte maturation [58]. ECM-binding peptides include heparin-binding peptide from antithrombin III, heparan sulfate binding peptide derived from laminin (AG73), basement membrane binder peptide and heparan sulfate binding region of placental growth factor 2 [58]. Interestingly, the modified hydrogels showed high deposition of ECM after follicular culture, suggesting de novo formation of the basement membrane. This could therefore represent a breakthrough optimizing the culture of enzymatically isolated follicles, since during the isolation procedure the integrity of the basement membrane of the isolated follicles becomes compromised, negatively impacting the cellular organization and survival during follicular culture [59]. In contrast to mechanical isolation, enzymatic digestion is also less laborious, yields higher number of follicles in a shorter time and is more suitable for highly stiffed tissues, such as the ovarian cortex [60]. Finally, the use of chemically-defined hydrogels as an alternative to animal-produced hydrogels, such as matrigel, will surely make the translation of these in vitro techniques into clinical applications more feasible.

A different approach that has gained attention in recent years is the generation of a bioengineered ovary using a decellularized ovary as scaffold. In contrast to hydrogels, the use of decellularized scaffolds offers the advantage of conserving the natural ECM, such as the precise collagen composition present in the tissue [61]. The decellularization is achieved by a combination of physical, chemical, and enzymatic treatments. To remove the cellular components from the ovary, the tissue is first incubated with detergents, such as sodium dodecyl sulfate (SDS), sodium lauryl ester sulfate (SLES) or Triton X-100. Next, enzymatic incubation with DNAse and RNAse follows to increase the biocompatibility of the scaffolds by reducing the immunogenicity [62]. Despite promising results, this technology is still in its infancy and more studies are necessary to validate its potential to promote folliculogenesis.

## 4. Ovarian Organoids to Study Ovarian (Somatic) Physiology and Disease

### 4.1. ECM Deposition and Fibrosis

The ovarian ECM undergoes constant and extensive structural remodeling during each and every ovulatory cycle, making the ovary one of the most dynamic organs of the human body. The activation and survival of primordial follicle is highly regulated by mechanical cues provided by the surrounding ECM; therefore, a deeper knowledge of the ECM composition could help overcome the challenges faced during the activation and growth of human primordial follicles in vitro. It is known that mechanical fragmentation of the ovarian cortex induces activation of unilaminar follicles [63,64]. This has led to the hypothesis that ECM remodeling during this procedure could trigger follicular activation. Del Valle and colleagues reported a significant increase in collagen IV in the stromal compartment of human ovarian cortex tissue cultured for 8 days, indicating that the ovarian cortex became more fibrotic during culture [51]. By contrast, Groibos and colleagues reported that human ovarian cortex tissue cultured for 6 days showed decreased collagen and increased elastin deposition [65]. This discrepancy may have resulted from differences in either the culture protocols or the donor material used. For example, Groibos and colleagues used ovarian cortex fragments freshly isolated from cisgender women and applied mechanical stretching before culture, whereas Del Valle and colleagues used cryopreserved-thawed human ovarian cortex tissue from transgender individuals without mechanical stretching. Interestingly, it has been reported that testosterone supplementation as a hormone-affirming therapy can lead to stiffer ovarian cortical tissue due to ECM accumulation [66].

To conclude, a study using mice neonate ovaries has also suggested a direct role for ECM in the regulation of dormancy/activation of unilaminar follicles [67]. By digesting the ovarian ECM using collagenase type IV and trypsin, the FOXO3 complex, a negative regulator of oocyte growth [68], translocated from the nucleus to the cytoplasm of the oocytes resulting in follicular activation. By contrast, when the pressure was increased by incubating mice ovaries in a pressure chamber, FOXO3 was observed in the nucleus of oocytes. This study suggested that ECM stiffness (compression) played a role in the cellular localization of FOXO3, a key factor in the balance between follicular dormancy and activation.

### 4.2. Hormonal Production and Menopause

The endocrine function of the ovary plays a major role in women’s health, as evidenced by the symptoms that accompany menopause. Menopause is caused by a decreased estrogen level, as consequence of the depleted ovarian follicular reserve. It often results in vasomotor symptoms (hot flashes and night sweats), urogenital atrophy, osteoporosis, cardiovascular disease and increased all-cause mortality [8,9,11,69]. Hormonal replacement therapy has become the standard treatment to improve the quality-of-life during and post menopause, but this treatment has been shown to increase the risk of breast cancer, thromboembolism, stroke and coronary artery disease [70]. One of the reasons for these side effects is that with hormonal therapy alone, the feedback mechanism of the hypothalamic-pituitary-ovarian axis is not in place. A solution for this could be to generate transplantable ovarian organoids containing hormone-producing cells, granulosa cells and theca cells, that could induce and react to pituitary-made gonadotropins.

Yoon and colleagues succeeded in the fabrication of ovarian organoids (or spheroids) that were able to restore the main endocrine ovarian roles after transplantation into ovariectomized rats [71]. The ovarian organoids were produced by aggregation of rat granulosa cells into spheroids, coating the spheroids with hydrogel to mimic the basement membrane and finally encapsulating the spheroids with rat theca cells. After transplantation of the ovarian organoids, endometrium regeneration and prevention of bone loss was observed [71]. These results support the idea that transplantable ovarian organoids could be promising as cell-based menopausal hormonal therapy. However, this technology was based on the large number of rat granulosa and theca cells, while in menopausal individuals the follicular reserve is basically depleted. This represents a big challenge for the isolation of autologous follicular somatic cells to create artificial follicles. An alternative approach could be to differentiate iPSCs from menopausal individuals into granulosa and theca cells in vitro and to subsequently generate transplantable ovarian organoids, but differentiation protocols to obtain both types of somatic ovarian cells in humans are still under development and hence not yet available for clinical applications [72].

### 4.3. Modelling Polycystic Ovary Syndrome (PCOS)

PCOS is a complex endocrine disorder that impacts female reproductive health [73,74]. The ovaries of PCOS women typically contain a high proportion of arrested antral follicles with dysfunctional granulosa cells, attributed to an imbalance in the ratio of pituitary hormones (FSH and LH), hyperinsulinemia and excessive androgen production [75,76]. Traditionally, animal models, such as dihydrotestosterone (DHT)-induced PCOS mice, or 2D culture of granulosa cells isolated from PCOS patients have been used to study the effects of elevated androgen levels on the ovary and specifically on the granulosa cells [77,78]. Recently, Liao and colleagues used ovarian organoids to investigate the metabolic effects of androgen on granulosa cells during follicular growth [79]. For this, alginate-encapsulated mouse secondary follicles were treated with 10 μM dehydroepiandrosterone (DHEA) to simulate the hyperandrogenic environment characteristic of PCOS. Their findings revealed that excessive androgen concentrations blocked follicular growth, cumulus expansion and ovulation in vitro [79]. These effects were associated with impaired steroidogenesis and reduced lipid metabolism in granulosa cells. Interestingly, flutamide, an antagonist of androgen receptor (AR), restored follicular growth [79], suggesting that AR may be a therapeutic target to treat the anovulatory effects of PCOS and showcasing the usefulness of ovarian organoids to understand ovarian dysfunction.

## 5. Ovarian Organoids for Cancer Research

### 5.1. Disease Modelling for Ovarian Cancer (OC)

**Figure 3:**
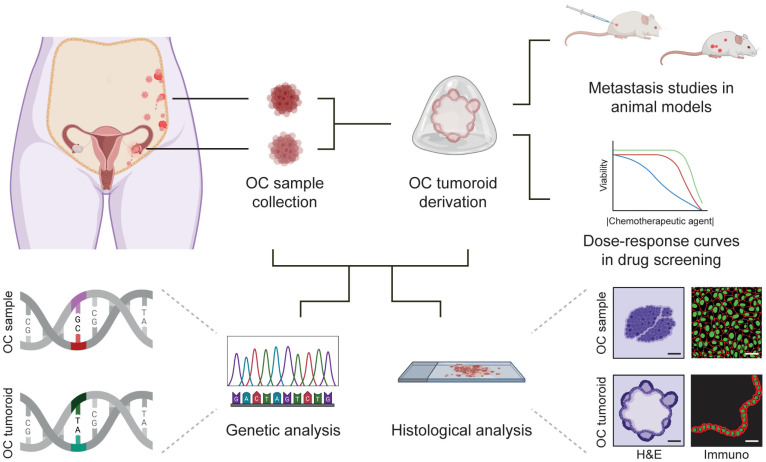
Establishment of ovarian cancer (OC) tumoroids, characterization and applications in personalized medicine. Biopsies of different OC types found in different body locations can be collected and used for OC tumoroid derivation. OC samples and OC tumoroids can be used for genetic analysis and histological analysis. Moreover, OC tumoroids can be used for metastasis studies in animal models or dose–response curves in drug screening assays (different lines represent different donors). Parts of this figure were created using https://www.biorender.com/ (accessed on 25 April 2023).

OC is a large group of heterogeneous cancers, characterized by a high index of chemotherapy resistance and poor survival [80], making it one of the deadliest gynecological malignancies and the eighth most common cause of cancer-related death among women (https://gco.iarc.fr/, accessed on 31 March 2023). The generation of patient-specific OC organoids can offer an important opportunity for disease modelling and personalized medicine (Figure 3). To generate tumor organoids (tumoroids) and, in particular, OC tumoroids from ovarian tumors, the tumor biopsies are dissociated into fragments or single-cells, embedded in hydrogel and cultured under specific conditions optimized per cancer type [81,82]. Kopper and colleagues, as well as Maenhoudt and colleagues, have reported the establishment of 56 and 13 OC tumoroid lines, respectively [81,82]. The culture media used in the two studies differed, but supplementation with R-spondin 1 (RSPO1) and Neuregulin 1 (NRG1) was used in both [81,82]. As expected, the derivation efficiency was variable, but OC tumoroids were obtained both from fresh and cryopreserved biopsies. OC tumoroids could be maintained long-term in culture, retained histological features and tumor characteristics, such as expression of TP53 and PAX8, and were used for genetic analysis [81,82].

Due to the anatomical proximity, OC can easily metastasize into the peritoneal cavity, penetrating the vascular system and, from there, leading to poor prognosis [83,84]. In order to attach and invade the peritoneum, OC cells need to go through an adaptive mechanism that enables cell adhesion and proliferation [83,84]. Although such mechanism is not fully understood, loss or switch in the orientation of the apical-basal polarity of epithelial cells has been linked to cancer initiation and metastasis [85]. Interestingly, tumoroids are also able to invert their apical-basal polarity (apical-in or apical-out) [86,87], and could therefore be a suitable model to investigate mechanisms of metastasis. OC tumoroids embedded in hydrogel (in contact with ECM) have apical-in polarity; however, OC tumoroids cultured in suspension (ascites) show an apical-out polarity [88]. OC tumoroids cultured in suspension showed a switch in polarity, from apical-out to apical-in, as early as 6 h post contact with hydrogel [88]. Kawata and colleagues further showed that inhibiting the polarity switch by applying SRC kinase family (SFK) inhibitors, such as dasatinib, prevented OC tumoroids from attaching to hydrogel and suppressed the number of peritoneal metastatic foci formed after injecting OC tumoroids in the peritoneal cavity of mice [88]. The use of OC tumoroids are providing new insights into the mechanism of peritoneal metastasis and ways to prevent it.

In humans, some cases of HGSC are characterized by peritoneal metastasis, whereas others only show the presence of OC [83]. Furthermore, the tissue of origin of HGSC (OSE-origin or FTE-origin) remains inconclusive. Studies applying peritoneal injection of engineered mouse OC tumoroids in mice to clarify the cell type of origin of HGSC have suggested a dual origin [89,90]. Mouse FTE-derived OC tumoroids were highly metastatic, compared to mouse OSE-derived OC tumoroids [89]. In agreement, in a proteomics study using 26 OC cell lines, Coscia and colleagues concluded that the two subtypes of HGSC may coexist, one FTE-derived and one OSE-derived [91]. OC tumoroids could become a preclinical platform to help clarify both tumor origin and metastatic mechanisms.

### 5.2. Drug Discovery and Drug Screening in Cancer Research

Organoid technology offers an unprecedent opportunity for drug discovery and personalized medicine [92]. Patient-specific OC tumoroids are becoming an important tool to study the genetics of tumor cells, to predict the response to different chemotherapeutical agents and to understand mechanisms that lead to chemotherapy resistance [93,94,95,96] (Figure 3). Importantly, in vitro drug response of patient-derived OC tumoroids seems to correlate to the patient’s clinical response, although intra-patient heterogeneity remains an issue [97]. An interesting innovation that resulted in decreasing OC manipulation while increasing screening speed was proposed by Phan and colleagues. They seeded OC tumoroids, established within 2–3 days and embedded in 10 μL hydrogel, on the rim of a 96-well (“ring gel”) [98] and achieved media change and drug treatment in about 2 min per 96-well plate [98]. In conclusion, OC tumoroids-based technology is becoming a valuable tool to assist clinical approaches for personalized medicine as well as to develop novel anti-cancer treatments.

## 6. Clinical Applications and Limitations

In contrast to the successful transplantation of ovarian cortex tissue in patients to restore fertility, there are still no clinical-grade protocols available to grow and mature oocytes present in ovarian cortex tissue in vitro, that could subsequently be used for reproductive purposes [2]. Once available, this technology will represent a tremendous breakthrough allowing a large group of female oncological patients (with blood-related cancer types), from whom it is not feasible or possible to isolate MII oocytes prior to therapy, to become biological parents later in life. The in vitro growth and maturation of follicles is dependent on follicular density, shows high donor-to-donor variation and, as a result, the culture protocols available are not robust and show low reproducibility [99,100]. A major limitation is that there are currently no protocols available to grow and mature oocytes present in ovarian cortex tissue in vitro starting from cryopreserved material, which represents the patient material available for clinical purposes.

In addition to a direct role in human reproduction, the creation of transplantable ovarian follicles from ovarian tissue could potentially play an important role in restoring the endocrine function of the ovary by regulating not only the ovarian hormonal levels, but also restoring the feedback mechanisms of the hypothalamic-pituitary-ovarian axis in individuals in menopause, with POI or that underwent oophorectomy. However, obtaining functional follicles is highly complex, requiring pivotal interactions between different follicular cell types, fine-tuning the matrix composition and stiffness, finding follicular somatic cells from the same donor or efficiently differentiating PSCs into functional granulosa and theca cells [72]. On that note, there are only a few patient-specific good manufacturing practice (GMP) quality iPSC lines worldwide, with defined quality and safe for transplantation. Generating GMP quality iPSCs is still very expensive and laborious, requiring specialized and certified GMP facilities [101]. The use of patient-specific lines in clinical trials is still limited [101] and it remains unclear whether patient-specific iPSCs will ever be considered safe enough for reproductive purposes by the regulatory agencies, such as the Food and Drug Administration (FDA) and the European Medicines Agency (EMA).

OC tumoroids are able to recapitulate aspects of the OC, such as the genetic makeup and the response to chemotherapeutical treatments. However, the heterogeneity found in OC tumoroids, both inter- and intra-donor, as well as genetic mutations acquired during prolonged culture, can jeopardize the reproducibility and scalability of the platform. Moreover, the complexity of organoids is still limited and not all cell types are present within this model, with immune cells usually being missed, meaning that the interaction between the immune system and tumor cells is lacking in vitro. Most importantly, protocols for OC tumoroids derivation must also become significantly shorter within the range of clinical decisions before this technology can be used for personalized medicine.

The generation of 3D ovarian organoids holds significant potential for applications in female fertility preservation, disease modeling, and drug testing. However, this technology still faces many challenges [19]. For example, starting from primary tissue may lead to a reduced number of viable cells or the loss of specific cell types after tissue dissociation. Tissue heterogeneity, such as variation in stromal cells, follicular density, innervation, immune cells and vascularization across different patients and ovarian regions, contributes to reduced morphological and functional reproducibility. Using stem cells (iPSCs or mesenchymal stromal cells) as starting point to make organoids also poses specific challenges, such as incomplete differentiation protocols, defective cellular compartmentalization and difficulties recreating tissue architecture. Finally, the use of organoids will not replace the use of experimental animals but can certainly contribute to a reduction and make the translation to human applications more straightforward [102]. Despite the current limitations, ovarian organoids seem a suitable platform for high-throughput drug screenings, accelerating the efficacy and toxicity tests for new drugs without risking the individual’s health.

## 7. Future Perspectives

We envision that the use of ovarian organoids from ovarian cortex tissue will have a major impact on the health and quality of life of females and human reproduction in general. The possibility to grow and mature MII oocytes in vitro from (dormant primordial follicles in) cryopreserved ovarian cortex tissue would be revolutionary, allowing any individual with an ovarian follicular pool to undergo fertility preservation, creating independence from the biological clock. This technology would allow females to become biological parents at any age, with the use of medically assisted reproduction (MAR). Moreover, this technology could also replace ovarian hormonal stimulation for oocyte retrieval during MAR, avoiding the need to expose individuals to high gonadotropic treatments that can lead to ovarian hyperstimulation syndrome. In addition, the use of OC tumoroids may pave the way to personalized medicine, helping with clinical decisions in finding adequate drug treatments, as well as understanding disease mechanisms. As bioengineered 3D ovarian models continue to evolve and improve, they will likely become an indispensable tool to understand diseases and develop new therapies adapted to female physiology and change the way we reproduce.

## Data Availability

Not applicable.

## Abbreviations

3D, three-dimensional; AR, androgen receptor; COC, complex-oocyte-cumulus; DHEA, dehydroepiandrosterone; DHT, dihydrotestosterone; ECM, extracellular matrix; EMA, european medicines agency; FDA, food and drug administration; FTE, fallopian tube epithelium; GCLCs, granulosa cell-like cells; GMP, good manufacturing practice; HGSC, high-grade serous ovarian cancer; iPSCs, induced pluripotent stem cells; MII, metaphase II; MAR, medically assisted reproduction; OC, ovarian cancer; OSE, ovarian surface epithelium; PCOS, polycystic ovary syndrome; PGCs, primordial germ cells; PGCLCs, primordial germ cell-like cells; PSCs, pluripotent stem cells; SDS, sodium dodecyl sulfate; SLES, sodium lauryl ester sulfate; TCLCs, theca cell-like cells.
